# Possibilities of Using 3D Printing with Polymers as Structural Components

**DOI:** 10.3390/ma18235384

**Published:** 2025-11-28

**Authors:** Marcin Artur Kwapisz, Piotr Paszta, Wiktor Lacki

**Affiliations:** 1 Faculty of Production Engineering and Materials Technology, Czestochowa University of Technology, Al. Armii Krajowej 19, 42-201 Częstochowa, Poland; 2 Faculty of Mechanical Engineering, Czestochowa University of Technology, Al. Armii Krajowej 21, 42-201 Częstochowa, Poland; piotr.paszta@pcz.pl (P.P.); wiktor.lacki@pcz.pl (W.L.)

**Keywords:** incremental methods, 3D printing, FDM/FFF, FEM analysis, strength testing, PLA, ABS, PA6, PA12

## Abstract

This article investigates the potential of using polymer-based Fused Filament Fabrication (FFF) 3D printing to manufacture structural components. This study aimed to explore the application of this technology within manufacturing processes. This research focused on material analysis, using a gear wheel from a lawn mower’s drive system as a case study. To achieve the purpose of this study, a reverse engineering process was carried out to create 2D and 3D documentation based on a physical piece. The geometric model created in the CAD environment was optimized for volume. It was intended to keep the material expenditure unchanged, which is intensified during the 3D printing process due to the need to apply an adhesive layer and supports. The final design process of the geometric model of the prototype was subjected to numerical analyses in terms of total deformation and reduced stresses for materials commonly used as filaments in the FDM/FFF 3D printing technology. The basic filaments PLA, ABS, and PA6 and PA12 were analyzed in this study. The results of the analyses showed that two of the four selected filaments had to be rejected due to significant deterioration in strength properties. Finally, the prototype workpieces were printed using materials approved for the manufacturing process.

## 1. Introduction

The household appliance market, among others, offers a wide variety of products within a single category. From the consumer’s perspective, the most common differentiating factor between models is price [1]. Unfortunately, the higher price of a device is not always accompanied by the quality of components and the manufacturer’s commitment to the spare parts market. This manifests itself in the form of unprofitable repairs due to the lack of available components, or their prices often exceed 70% of the purchase price of a new device [2].

The development and increasing ubiquity of 3D printing technology offer new, cost-effective possibilities for producing small-batch, custom-made spare parts. Until now, making a part from a composite or polymer material required advanced machinery or was not plausible economically. Currently, to produce a functional workpiece, it is theoretically enough to have basic measuring instruments, a computer and a 3D printer. It is obvious that the quality of such a workpiece depends on the accuracy of the instruments and the device, but it is still a solution that is cheaper by an order of magnitude. In such a situation, a reduction in quality can sometimes be considered justified [1,2].

Most manufacturers only provide spare parts during the warranty period; after that time, they are no longer available, requiring the purchase of a new device and disposal of the existing one. This work attempted to recreate a damaged part of a gear transmission. The analyzed gear wheel is a wear-and-tear component and acts as a safety device in the event that the mower blade encounters a hard object. Based on reverse analysis and numerical methods, the recreated gear wheel was prepared. The numerical analysis allowed for the selection of a suitable 3D printing material that would match the strength properties of the original component while maintaining all functions, including the safety required by the analyzed gear wheel [2,3,4].

The selected gear is responsible for transmission of power from the engine to the chassis of a lawnmower from the manufacturer NAC. The choice of product was mainly influenced by the manufacturer’s approach to updating its model range over the years. This manufacturer changes the model range every season, and the aftermarket only has parts that fit the last two generations.

## 2. Materials and Methods

### 2.1. FDM 3D Printing Technology

3D printing is still considered an emerging technology that facilitates many modern and innovative solutions. The main advantage of 3D printing is the simplicity of obtaining complex geometries compared to other manufacturing technologies, such as injection molding or cavity methods. An additional advantage is the lower price of professional printers relative to production machines with the required accessories. The disadvantage that prevents 3D printing from being used in mass production is the long processing time required to acquire a workpiece. With a combination of advantages and disadvantages in commercial realities, 3D printing has become an ideal solution for the engineering centers that are responsible for product development. The production of a prototype, by producing a single unit, which requires confirmation of functionality in reality, is most economically justified when using the above-mentioned technology. Currently, there are many variants of 3D printing technologies offered on the market, from basic ones, such as deposition modeling (FDM), to advanced processes such as directed energy deposition (DED) technology, in which the overlay material is fused using a laser beam [2,3,4].

The most popular 3D printing technology is the previously mentioned FDM/FFF technology. Its prevalence is most influenced by the simplicity of the process and its neutrality to the environment. In simple terms, it is based on melting a material from a reel, called filament, in a suitably adapted head, and then laying tracks of melted material on a heating table [5,6].

**Figure 1:**
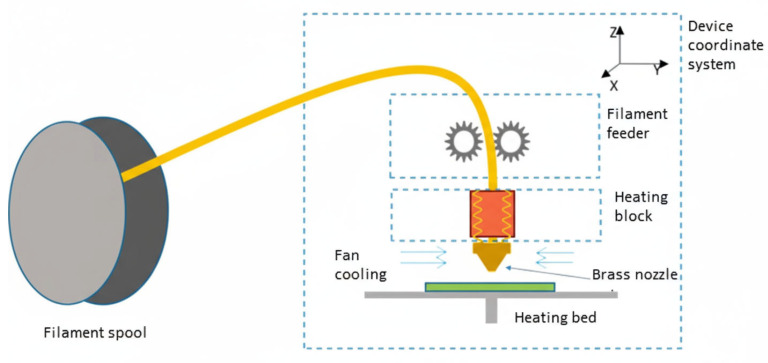
Schematic diagram of a 3D printer using FDM technology [7].

The filament is distributed to the head via an extruder. Two types of layouts are encountered: in one, the feeder is located just above the filament reel. The second solution, where the feeder is located just above the head, is shown in Figure 1. This solution has many advantages as well as disadvantages.

The main advantage is the ability to use flaccid filaments, such as rubber ones. The feeder mounted above the reel is unable to deliver such filament at the appropriate speed through the long feed tube (usually in the form of Teflon tubing). The disadvantage of placing the feeder just above the head is the additional inertial load on the XY system, resulting in the need for more durable, yet more precise drives for the respective axes. The feeder placement above the head is usually found in more expensive equipment [3,4,5].

The head consists of a heating block assembly, a cooling fan and a nozzle, usually made of brass. The material supplied through the feeder is melted in the channel of the heating block, after which it is pressed through the nozzle in the form of a thin cylinder of the diameter given by the nozzle. Various nozzle diameters are available on the market, but 0.4 mm nozzles are most commonly used. Another parameter that the user can influence is the height of the path. It is defined programmatically. The higher the path height, the faster, but also less accurate, the printout. The situation is analogous for nozzle diameters. Depending on the material used, the path may need to be cooled quickly to improve print quality. For this purpose, a cooling fan is provided in the head structure. The user usually also has an influence on its speed, most often defined as a percentage [6,8,9].

When selecting parameters, the user must also specify the filling of the printed workpiece. This includes the thickness of the outer wall of the workpiece and the type of filling (including its density expressed as a percentage) [10].

### 2.2. Filaments Used in the FDM Technology/FFF

FDM/FFF technology, which uses the starting material in the form of a filament wound on a reel, called a filament, in theory is capable of using any material that goes from a solid state to a liquefied state under a certain temperature, and then returns to a solid state after cooling. However, practice shows that not all materials are suitable for this technology. The main obstacle turns out to be the melting temperature of the material. On average, printers on the market are able to maintain a stable temperature of the heating block of no more than ca. 300 °C. Materials that require higher temperatures are automatically reserved for professional equipment. Fortunately for the users of 3D printers using FDM technology, despite the limitations that exist, the range of materials available on the market is impressive, with manufacturers launching more each year [4,5,6,11].

The filaments most commonly used in the FDM technology include:

- PLA (polyactide) is classified as a filaments that is easier for printing, especially recommended for those starting out in 3D printing. It is a biodegradable material derived from corn or potato meal. It is not a toxic material; so, the printed workpieces can come into contact with food. No gases are emitted during printing, making it possible to set up the printer, e.g., in an office space [12]. The relevant properties in terms of 3D printing and the strength of the printed parts are shown in Table 1.
- ABS (acrylonitrile-butadiene-styrene terpolymer) was one of the first materials used in 3D printing. This is due to the widespread use of this material in injection molding technology. It is extracted using substrates derived from petroleum refining; so, it is not advisable for it to come into contact with food. It exhibits good wear resistance and strength at high temperatures. In the 3D printing process, it is one of the more challenging materials, as high internal stresses are created due to the path cooling too quickly. The impact of such stresses is the detachment of the workpiece from the work table or the lack of adhesion among layers. As the material heats up, an irritating odor is emitted in the print head, which precludes printing, for example, in office areas [12]. Its properties are shown in Table 1. The relevant properties in terms of 3D printing and the strength of the printed parts are shown in Table 1.
- PA (Polyamides) are very rarely used as pure filaments in their natural condition. The most common filaments are those reinforced for specific applications, e.g., PA6GF30—a filament with 30% glass fiber by volume. The most popular pure polyamides are the materials designated as PA6 and PA12. The main features are good resistance to chemical reagents and abrasion resistance, with PA12 showing better properties in these categories than PA6. The materials are also characterized by high hygroscopicity, which makes it advisable to dry the filament before each printing and store it in special packaging [13]. The relevant properties in terms of 3D printing and the strength of the printed parts are shown in Table 1.

## 3. Results and Discussion

### 3.1. Geometry Design in the CAD Environment

**Figure 2:**
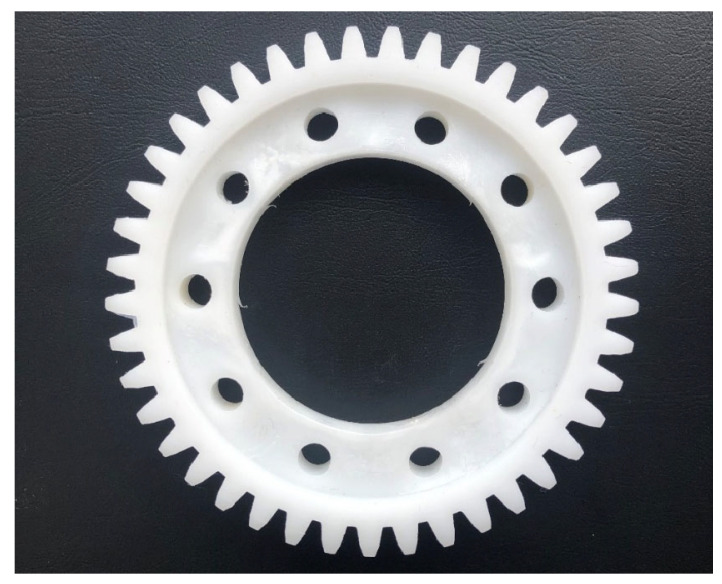
Base workpiece used in the tests.

In accordance with the adopted purpose of this paper, a gear wheel dedicated to the transmission of the drive system of a petrol lawnmower from the manufacturer NAC was used as an example of a production workpiece, on the basis of which the tests were carried out. The model of the lawn mower for which the gear wheel was designed, is W510VY. The workpiece obtained from the manufacturer is shown in Figure 2. For the purposes of preparing two-dimensional and three-dimensional technical documentation, the Autodesk-Inventor Professional 2025 distribution CAD program was used.

### 3.2. Preparation of the Base Model

**Figure 3:**
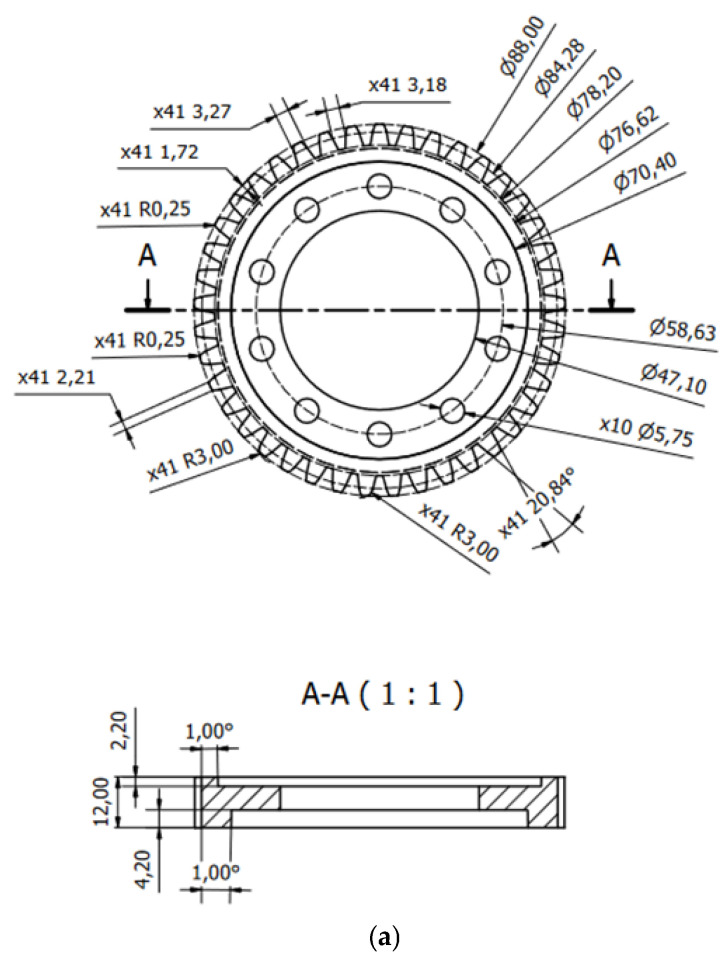

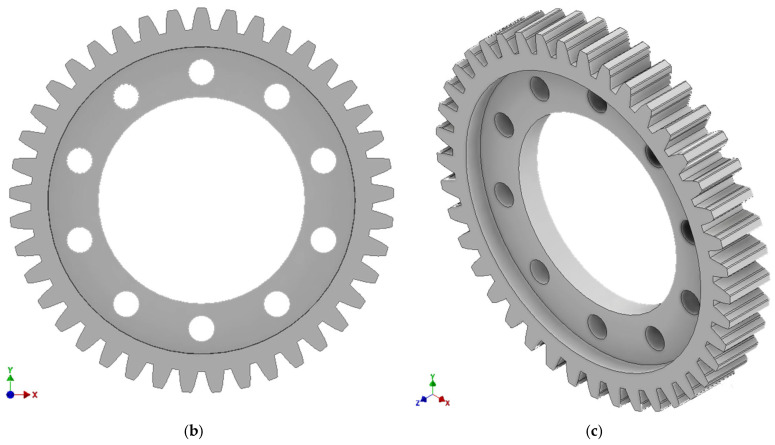
Base geometric model: (**a**) dimensions [mm], (**b**) front view, (**c**) spatial view.

Preparing the base model in a CAD environment involved measuring the necessary dimensions. The accuracy of the measuring instrument was ±0.01 mm. Each dimension was measured five times, and then the calculated average value was taken as the measurement value. During the measurements, significant dimensional discrepancies of theoretically symmetrical elements, such as teeth, were noted, as a result of which the gear’s manufacturing accuracy class was determined to be in the range of class 8 to class 12. Based on the obtained measurement values, a geometric model of the base workpiece was prepared (Figure 3a–c). In addition, two-dimensional technical documentation was prepared based on the three-dimensional model.

### 3.3. Numerical Analysis of the Base Model

With the geometry of the base model prepared in the CAD environment, it was possible to perform numerical analysis to determine the key strength values under a specific load. Polyoxymethylene (POM) was determined as the material from which the gear purchased from the manufacturer was made. The material properties were defined in Ansys Mechanical 2021 R1. Second-order tetrahedral (Quadratic) elements, known in the APDL nomenclature as SOLID187 (10-node), were used. The choice of second-order elements, which have additional nodes on the edges, was crucial. These elements significantly better reproduce complex geometric curves (such as the involute profile of a tooth) and are able to accurately capture the high stress gradients expected in concentrated areas, such as at the tooth root and around holes. Using linear (first-order) elements would have produced results with underestimated stress values and falsely higher stiffness.

**Figure 4:**
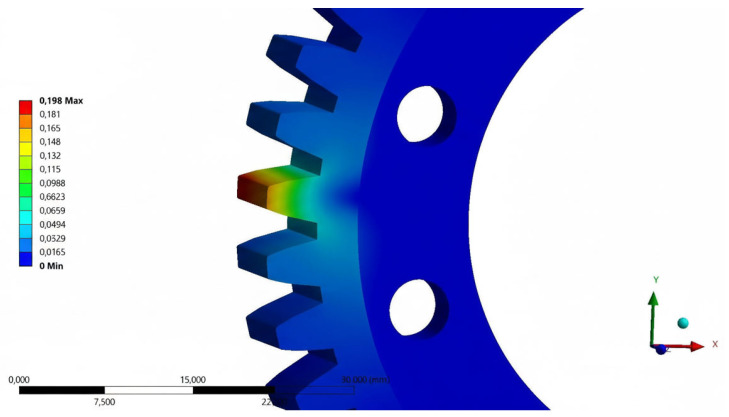
Results of total strain tests [mm]—loading force of 600 N, POM material.

**Figure 5:**
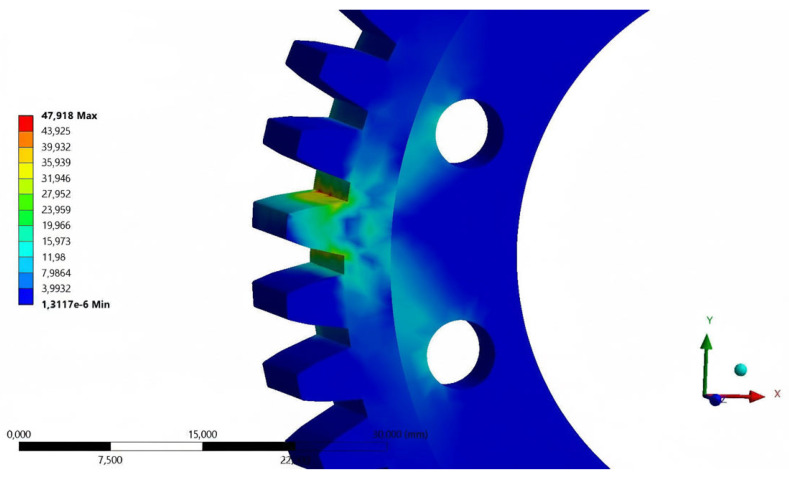
Results of reduced stress tests [MPa]—loading force of 600 N, POM material.

Preparation of the numerical model began with defining the boundary conditions. Receiving all degrees of freedom was assigned to the inner surfaces of the holes intended for the screw connection of the gear to the drive hub of the mechanism. A static load of 600 N was assigned to the working surfaces of one of the teeth in one possible direction of rotation of the gear wheel. Applying all the pressure to a single tooth is the most critical strength test for gear geometry. During rotary motion, the simulated condition lasts for a very short period of time, depending on the rotational speed, but combined with the number of repetitions of several hundred thousand, it will cause the greatest fatigue wear [14,15,16,17,18]. Two strength aspects were analyzed: total strain (Figure 4) and reduced stress (Figure 5). A global element size (in the Mesh -> Sizing -> Element Size section) of 1.0 mm was used in the analysis. Given that the maximum reduced stresses occur at the tooth root, local mesh refinement (Mesh Control Sizing) was applied. Critical surfaces, such as the tooth root fillets and the edges of the mounting holes, were locally dimensioned with a maximum element size of 0.2 mm. This ensured the presence of at least a few elements in the stress concentration zone, which is necessary to correctly capture the peak value. A mesh convergence study was conducted to validate the numerical model. A series of analyses were performed, systematically increasing the mesh density, reducing the global element size, and monitoring the key output parameter, the maximum von Mises stress. It was found that after reaching less than 100,000 elements, further mesh refinement resulted in acceptably small changes in the results. This confirmed that the obtained results were independent of the discretization density and that the adopted mesh of 97,960 elements was suitable for the analysis.

The results obtained during the first analysis in comparison with the material data, showed the possibility of increasing the value of the loading force. Based on the material tables, the yield strength of the POM material is in the range of 60 to 70 MPa. Taking into account the safety factor of 80%, the stresses reduced during the analysis can reach the limit of 48 MPa (for a yield strength of 60 MPa). After trial analyses, the value of the safe static load for further calculations was assumed to be 600 N. The analysis of the base model was repeated for total strain and reduced stress. The total strain results of the loaded tooth geometry were used as a reference for the process of designing a prototype gear intended for the manufacturing process using 3D printing technology.

### 3.4. Prototype Design in the CAD Environment

At the beginning of the design process, criteria were adopted to determine the cost-effectiveness and release of the part for production using 3D printing technology:

1. the percentage reduction in volume must be greater than the percentage weakening of the workpiece in terms of strength properties,
2. the percentage weakening of stiffness of the workpiece must not exceed 10%.

The definition of the first requirement was dictated by the need to use supports and an adhesive layer that are typical for the FDM/FFF 3D printing technology. The reduced volume will help compensate for the material expenditure, as a result, in terms of material consumption, the process will not generate losses. The second requirement ensures preservation of the proper operation of the gear for which the gear wheel was designed. Greater stress of the tooth geometry will reduce the quietness of the system, tooth load misalignment and faster wear of the gear wheel [14,15,16,19,20,21].

**Figure 6:**
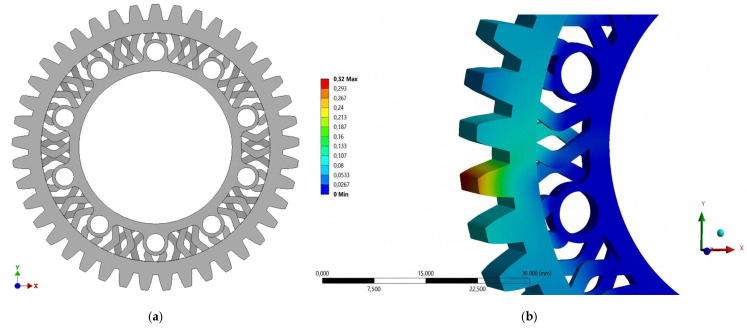
Geometric model of prototype No. 1 (**a**) front view, (**b**) result of total strain tests [mm]—loading force of 600 N.

The design process resulted in 3 prototype geometries. The first prototype used a spoke design in the center of the gear wheel (Figure 6a). Despite a volume reduction of 15.9%, the prototype was rejected due to a 68% increase in total strain of the tooth geometry (Figure 6b).

**Figure 7:**
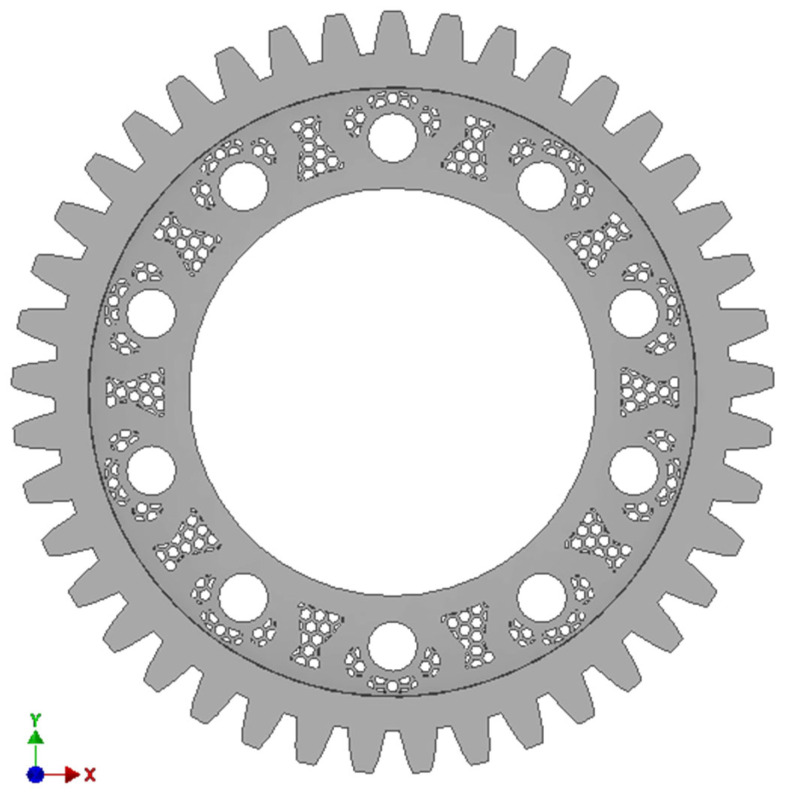
Geometric model of prototype No. 2—front view.

The second prototype geometry used a honeycomb lattice structure (Figure 7).

**Figure 8:**
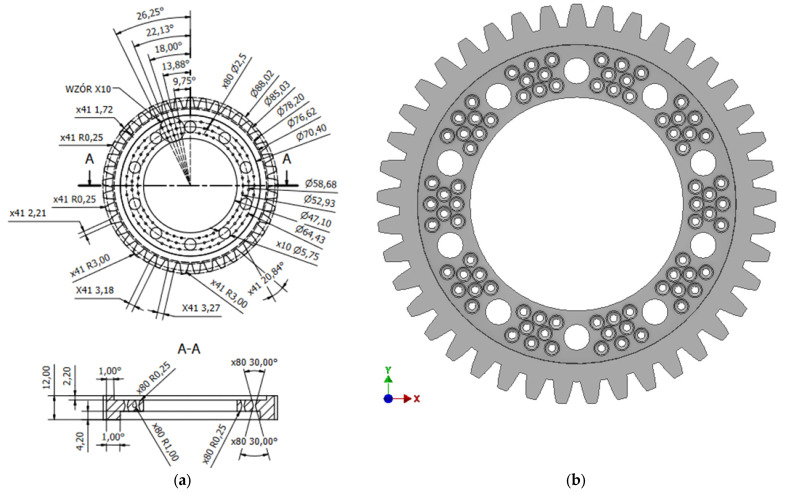
Geometric model of prototype No. 3: (**a**) dimensions [mm], (**b**) front view.

This solution resulted in a 5% reduction in volume and a 10.5% increase in total strain. The volume of the final prototype geometry was reduced by introducing additional holes in the center of the gear wheel (Figure 8).

**Figure 9:**
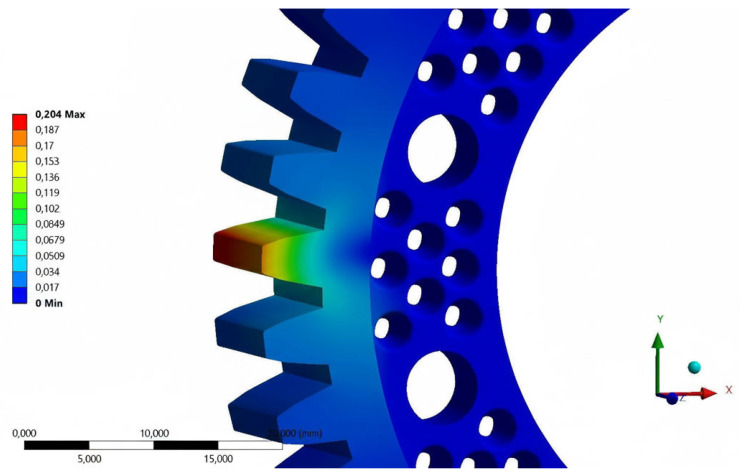
Analysis results of the tests for the total strain of prototype No. 3 [mm]—loading force of 600 N.

The resulting volume reduction was 6.6%, while the increase in total strain was 3% (Figure 9).

### 3.5. Analysis of the Results Obtained in the FEM Prototype Model

The successful design stage allowed us to conduct further numerical analyses. The necessary material data for numerical analysis of an object loaded with a static force was taken from the material tables. The properties were defined in the Ansys Mechanical 2021 R1 software environment for the ABS, PLA, PA6 and PA12 materials.

Developing a mathematical model for analyzing the prototype geometry using different materials in the Ansys Mechanical 2021 R1 software environment began with determining the geometry restraint surface. Receiving all the degrees of freedom was assigned to the surfaces of technical holes, analogous to the mathematical model used when comparing the prototype’s geometric model proposals to the base model (Figure 3). The static force loading the geometry was defined in the same way as in the previous analyses. Maintaining an identical restraint and loading arrangement provided the opportunity to compare the results of the respective analyses without casting doubt on reliability of the conclusions. Numerical analyses for the respective materials were carried out in two quantities: total strain and reduced stresses in the order of the material data presented.

### 3.6. Analysis of Numerical Results

The results of the analysis of the total strain of the tooth geometry of the prototype gear made of the ABS material relative to the one made of POM showed a gain of 32.3%. The increase in strain relative to the base gear made of POM is 36.5%. The maximum values of reduced stress in the tooth base area exceeded the yield stress threshold of the material used.

**Figure 10:**
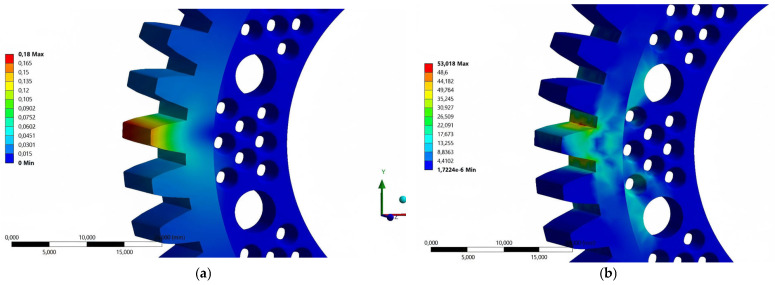
Test results for total strain–material: PLA, loading force: 600 N; (**a**) [mm], (**b**) [MPa].

Analysis of the total strain (Figure 10a) of geometry of the prototype gear made of the PLA material showed a reduction in maximum stress both compared to the prototype model made of POM and to the base model. Compared to the prototype geometry, the reduction is 11.4% while relative to the base geometry, it is 8.7%. A consequence of the higher stiffness of the geometry was the increased values of the reduced stresses obtained (Figure 10b) compared to the results of the ABS material analysis.

The results of the total strain analysis of the prototype gear geometry made of the PA6 material do not differ significantly from the results obtained for the base geometry of the POM material. To be exact, a reduction in maximum stress of 3.1% has been shown. Compared to the results of the prototype geometry made from the POM, the reduction was 6.1%. The obtained maximum values of reduced stresses are comparable with the results of the analysis for the PLA material.

The analysis conducted for the PA12 material showed the worst results for the maximum total strain of the tooth geometry compared to all the previous analyses. Compared to the prototype model of the gear made of POM, a gain of 111.1% was shown. The increase relative to the values obtained for the base model made from POM was 117.7%. The obtained values of maximum reduced stresses were lower than the values obtained in the analyses of the PA6, PLA and ABS materials.

### 3.7. 3D Printing

The final stage of the works was production of prototype parts from the selected materials. A semi-professional 3D printer, the Bambu Lab X1 Carbon, was used for this purpose. The choice of device resulted from the idea to test the latest solutions available on the market. Compared to FDM technology printers offered by competitors, Bambu Lab has built-in artificial intelligence modules that monitor print quality in real time using a vision system. This solution allows for an increase in production efficiency, reduces material losses and shortens the time of printing the part [17].

**Figure 11:**
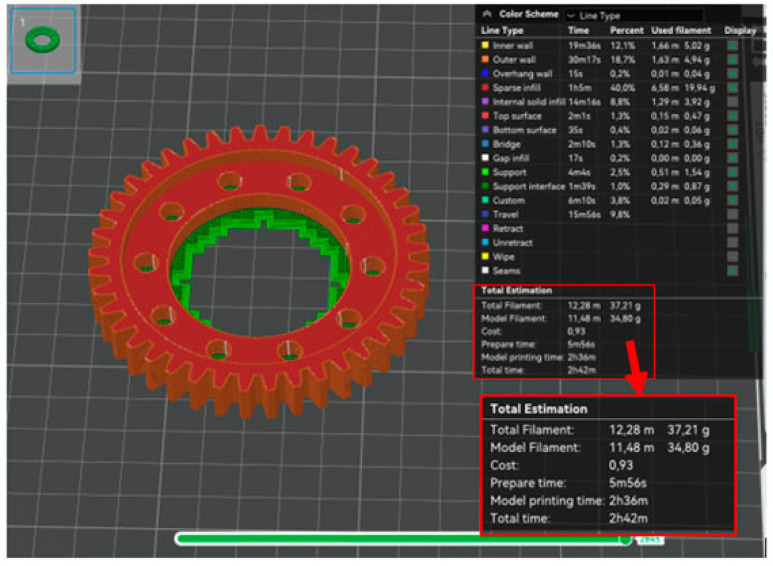
Summary of the program for printing the base model from PLA material.

**Figure 12:**
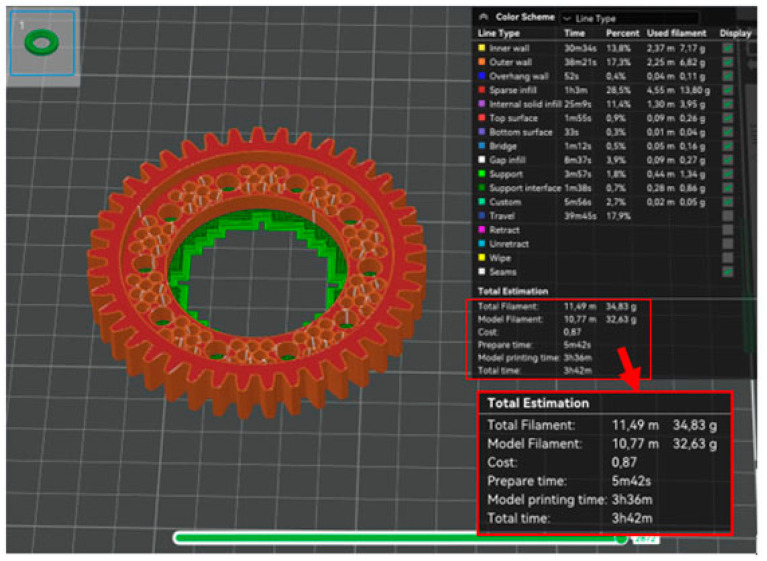
Summary of the program for printing the base model from PLA material.

**Figure 13:**
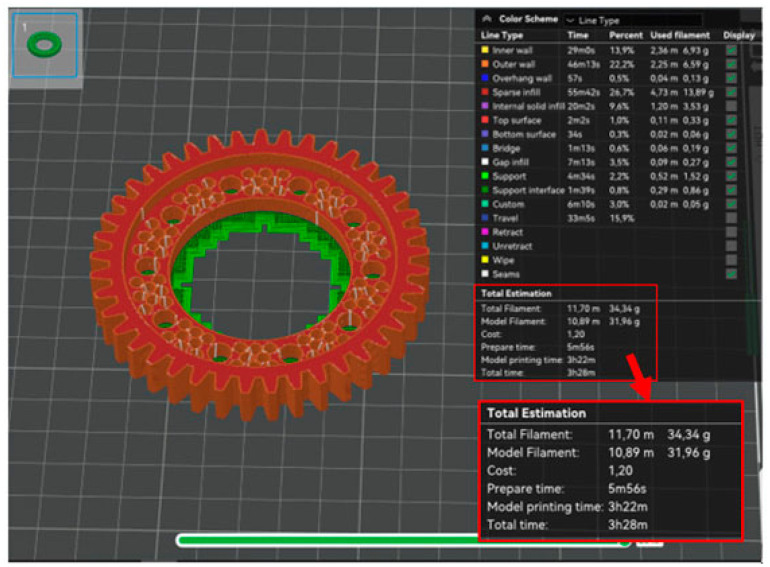
Summary of the program for printing the base model from PA6 material.

The first phase of the prototype printing process was to generate programs for the printer in the dedicated Bambu Studio V.2.2.2.56 software provided by the printer manufacturer. Three programs were generated: a program to print a base model using the PLA material (Figure 11), a program to print a prototype model using the PLA material (Figure 12), and a program to print a prototype model using the PA6 material (Figure 13).

Generating a program to print the base model only served as a benchmark. A comparison of the program for the base model and the program for the prototype model using the PLA material confirmed the success of the optimization process. In Table 2 contains a summary of the volume, weight and printing time of the analyzed product

The predicted weight of the prototype model differs slightly from the predicted weight of the base model without taking into account the supports. The comparison of the prototype model printing program using the PLA material and the prototype model printing program using the PA6 material demonstrates there are slight discrepancies in the predicted printing time and the final weight of the workpiece. Thus, the effect of material type on the energy intensity and material consumption of the manufacturing process was not demonstrated.

**Figure 14:**
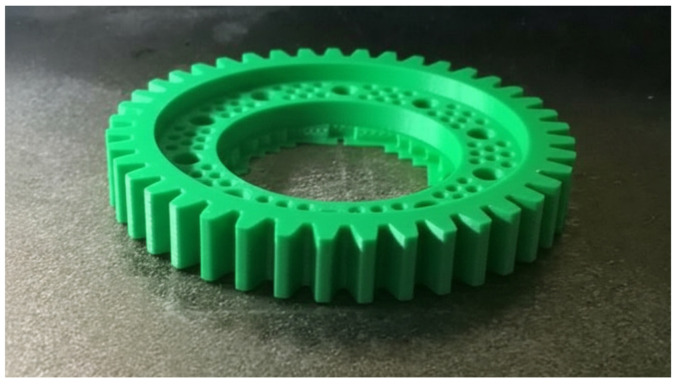
Prototype workpiece printed from the PLA material.

**Figure 15:**
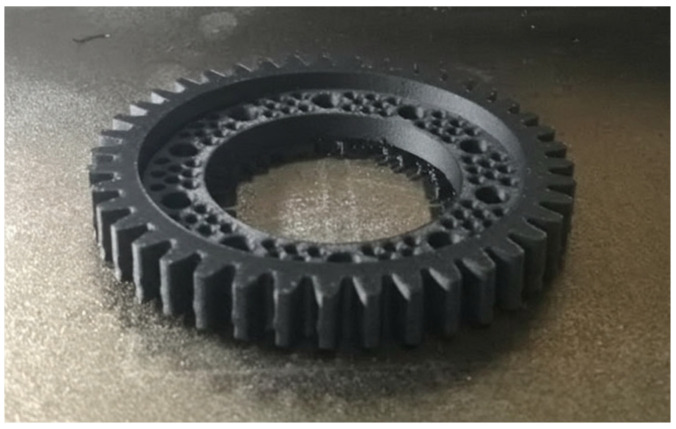
Prototype workpiece printed from the PA6 material.

Phase two of the prototype workpiece printing process involved uploading the previously prepared programs to the printer, loading the appropriate materials into the printer’s cartridge, and starting the printing process. After executing the programs, two prototype parts were obtained: one produced from the PLA material (Figure 14) and one from the PA6 material (Figure 15).

Comparison of the manufactured parts showed better quality of the prototype part made of the PLA material. Despite the differences in quality between the workpieces, in terms of geometry correctness, both printed workpieces were qualified as fit for use. In addition, a higher repeatability of the tooth geometry profile has been demonstrated compared to the base geometry purchased from the manufacturer.

## 4. Conclusions

The use of reverse engineering requires engineering knowledge in the stages of operating measuring instruments, preparing the appropriate CAD model, selecting the 3D printing material, and the printing process itself. Each of these stages requires human knowledge and experience [1,3,4,8,22,23].

The obtained results of numerical simulations confirmed the validity of performing this type of testing before the production of prototype parts. Based on these, of the four nominated materials, only two were approved for further production: PLA and PA6. The ABS material was rejected for two reasons: the threshold of 10% discrepancy of the maximum values of the total deformation of the tooth geometry was exceeded, and the maximum values of the reduced stresses exceeded the yield strength of the material, as a result of which the deformation of the tooth geometry would be fixed. Based on the results obtained, the PA12 material was rejected due to an increase of more than 100% in the values of maximum total deformation. In such a situation, it is reasonable to assume that teeth jump between each other during increased loading, which is categorically unacceptable. Conducting numerical analyses has significantly saved the potential capital provided for purchasing the materials and shortened the time required to print the parts. In addition, analysis of the results obtained for the qualified materials made it possible to define the first choice material, which was PLA. The gear made of PLA achieved the best strength properties; this material is biodegradable and is one of the cheapest on the market.

After final verification of the results of the examination conducted, it was possible to consider the purpose of this paper as fulfilled. In this article, it was demonstrated that the strength properties of the prototype geometry made of a material different than that intended by the manufacturer were met and even improved. The developed geometry with reduced volume of the prototype part allowed for the use the FDM 3D printing technology while maintaining the material expenditure for production of one workpiece.

## 5. Summary

The conducted research presented in this paper led to the following statements and conclusions:

- Despite their flaws due to the use of mainly theoretical material data, numerical analyses provide a reliable estimate of the validity of using a particular material in the production process.
- FDM 3D printing technology is a technology ever-evolving in all fields of technology, which in the future may make it the only viable manufacturing technology for low-volume production of workpieces.
- The performed optimization of the volume of the prototype gear geometry would not be economically justified for production using injection molding technology. Adding 80 holes would increase the cost of the injection mold to such an extent that the reduced material expenditure and associated capital savings would not be able to cover the investment in dies.
- Printing physical prototypes with high quality and reproducible tooth geometries from selected materials conclusively confirmed the feasibility of using 3D printing in part production.
- The use of PLA in further production as a base material was justified during tests in terms of both strength and production. Economically, the PLA material is also a better choice than PA6.
- During the production process, the additional advantage of being able to produce a single unit without trial prints was demonstrated. The prerequisite is to follow the proper preparation procedures recommended by the 3D printer manufacturer and to properly prepare the geometry of the printed workpiece.
- The ultimate form of confirmation of the results of this paper would be to conduct a load experiment involving mounting the prototypes in a dedicated gearbox, and then comparing actual wear against the base workpiece after a certain number of hours of operation.

## Figures and Tables

**Table 1:** Properties of selected filaments [8,9,13].

Parameter	Material			
	PLA	ABS	PA6	PA12
Printing parameters				
Heating block temperature [°C]	210	255	270	265
Working bed temperature [°C]	60	100	90	110
Printing in a thermal chamber	NO	YES	YES	YES
Inert to solvents	YES	NO	YES	YES
**Mechanical properties**				
Tensile strength upon break [MPa]	40–60	36–47	61	45
Impact strength [J/m^2^]	13–16	30–36	23	20
Deflection temperature under load [°C]	50–58	81–95	135	135

**Table 2:** Summary of the volume, weight and printing time.

	Volume [cm^3^]	Mass [g]	Printing Times
Base model POM material	28.173	39.724	-
Prototype POM material	26.313	33.42	-
Base model PLA material	28.173	34.80	2 h 36 m
Prototype PLA material	26.313	32.63	3 h 36 m
Prototype PA6 material	26.313	31.96	3 h 28 m

## Data Availability

The original contributions presented in this study are included in the article. Further inquiries can be directed to the corresponding author.
